# Genetic Analysis of the Role of Proteolysis in the Activation of Latent Myostatin

**DOI:** 10.1371/journal.pone.0001628

**Published:** 2008-02-20

**Authors:** Se-Jin Lee

**Affiliations:** Department of Molecular Biology and Genetics, Johns Hopkins University School of Medicine, Baltimore, Maryland, United States of America; Ecole Normale Supérieure de Lyon, France

## Abstract

Myostatin is a secreted protein that normally acts to limit skeletal muscle growth. As a result, there is considerable interest in developing agents capable of blocking myostatin activity, as such agents could have widespread applications for the treatment of muscle degenerative and wasting conditions. Myostatin normally exists in an inactive state in which the mature C-terminal portion of the molecule is bound non-covalently to its N-terminal propeptide. We previously showed that this latent complex can be activated *in vitro* by cleavage of the propeptide with members of the bone morphogenetic protein-1/tolloid (BMP-1/TLD) family of metalloproteases. Here, I show that mice engineered to carry a germline point mutation rendering the propeptide protease-resistant exhibit increases in muscle mass approaching those seen in mice completely lacking myostatin. Mice homozygous for the point mutation have increased muscling even though their circulating levels of myostatin protein are dramatically increased, consistent with an inability of myostatin to be activated from its latent state. Furthermore, I show that a loss-of-function mutation in *Tll2*, which encodes one member of this protease family, has a small, but significant, effect on muscle mass, implying that its function is likely redundant with those of other family members. These findings provide genetic support for the hypothesis that proteolytic cleavage of the propeptide by BMP-1/TLD proteases plays a critical role in the activation of latent myostatin *in vivo* and suggest that targeting the activities of these proteases may be an effective therapeutic strategy for enhancing muscle growth in clinical settings of muscle loss and degeneration.

## Introduction

Myostatin is a transforming growth factor-ß (TGF-ß) family member that plays a critical role in regulating skeletal muscle mass [1]. Genetic studies in mice [2], cattle [3]–[6], sheep [7], dogs [8], and humans [9] have shown that myostatin normally acts to limit muscle mass. Mice engineered to lack myostatin have about a doubling of skeletal muscle weights throughout the body, which results from a combination of increased fiber numbers and muscle fiber hypertrophy [2]. Hence, myostatin appears to play at least two distinct roles in regulating muscle mass, one to regulate the number of muscle fibers that are formed during development and a second to regulate muscle fiber growth. Based on this latter function, there has been considerable interest in developing agents capable of inhibiting myostatin activity for both agricultural and human therapeutic applications. In this respect, several engineered and endogenous myostatin binding proteins have been demonstrated to be capable of increasing muscle growth when administered to normal and/or dystrophic mice [10]–[14].

Like other TGF-ß family members, myostatin is synthesized as a precursor protein that is cleaved by furin proteases to generate the active C-terminal dimer. When produced in Chinese hamster ovary cells, the C-terminal dimer remains bound to the N-terminal propeptide, which maintains myostatin in a latent, inactive state [12], [15]–[16]. Most, if not all, of the myostatin protein that circulates in the blood also appears to exist in an inactive complex with a variety of proteins, including the propeptide [17]–[19]. We showed previously that the latent form of myostatin can be activated *in vitro* by dissociating the complex with either acid or heat treatment [12], [17] or by proteolytic cleavage of the propeptide with members of the BMP-1/TLD family of metalloproteases [12]. Here, I present genetic evidence suggesting that proteolytic cleavage of the propeptide may be the primary mechanism by which latent myostatin is activated *in vivo*.

## Results

### Generation and analysis of mice with a protease-resistant form of the propeptide

In previous studies, we showed that the purified complex of myostatin propeptide and C-terminal dimer can be cleaved at aspartate residue 76 by members of the BMP-1/TLD family of metalloproteases and that cleavage at this site in the propeptide results in activation of the latent complex [12]. Furthermore, we showed that changing this aspartate residue to alanine (D76A) affected neither the ability of the propeptide to form a complex with myostatin nor the ability of the latent complex to be activated by heat treatment but completely blocked the ability of the complex to be cleaved and activated by members of the BMP-1/TLD family. Finally, we showed that unlike the wild type propeptide, a form of the propeptide containing the D76A amino acid change could cause significant muscle growth when administered to mice.

**Figure 1:**
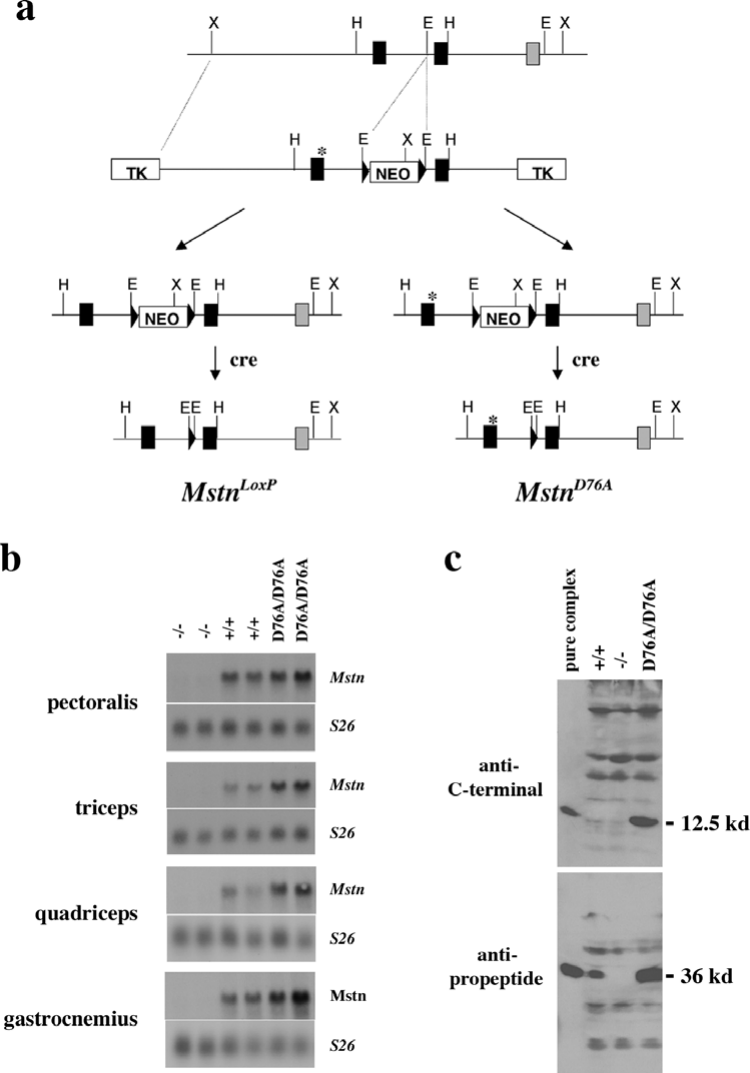
Generation of mice carrying a *Mstn* point mutation rendering the propeptide resistant to cleavage by BMP-1/TLD proteases. (a) Gene targeting strategy. Black and stippled boxes represent coding exons for the propeptide and C-terminal domain, respectively. The location of the point mutation (D76A) is denoted by an asterisk, and LoxP sites are denoted by triangles. Removal of the neo cassette using EIIa-cre mice resulted in *Mstn* alleles containing a single LoxP site (in intron 1) either with (*Mstn^D76A^*) or without (*Mstn^LoxP^*) the point mutation. (b) Northern analysis of *Mstn* RNA expression in mutant mice. Muscle RNA isolated from *Mstn^−/−^*, wild type, and *Mstn^D76A/D76A^* mice was electrophoresed, blotted, and hybridized with a *Mstn* probe. The blots were re-hybridized with a probe for the S26 ribosomal protein to control for loading. (c) Analysis of myostatin protein in mutant mice. Hydroxylapatite-bound serum samples isolated from wild type, *Mstn^−/−^*, and *Mstn^D76A/D76A^* mice were electrophoresed, blotted, and probed with antiserum directed against either the C-terminal domain [2] or the propeptide [15]. In each gel, the first lane contains purified myostatin latent complex isolated from Chinese hamster ovary cells [15].

To determine whether this mechanism for activating latent myostatin functions *in vivo*, I generated mice in which the aspartate to alanine change in the propeptide was introduced into the germline. As shown in Figure 1a, the D76A point mutation was engineered into a myostatin genomic construct, which was used for homologous targeting in embryonic stem cells. Following injection of the targeted embryonic stem cells into blastocysts, I obtained mice that transmitted the mutant allele through the germline. These mice were then crossed to EIIa-cre transgenic mice [20] to remove the neo cassette that had been placed into the first intron. In order to rule out the possibility that any observed effects might result from the LoxP site remaining following removal of the neo cassette, I also generated mice carrying this LoxP site but which were wild type at aspartate 76. Both of these lines were backcrossed at least 6 times to C57 BL/6 mice prior to analysis, and all analysis was carried out on both female and male mice at 10 weeks of age.

**Figure 2:**
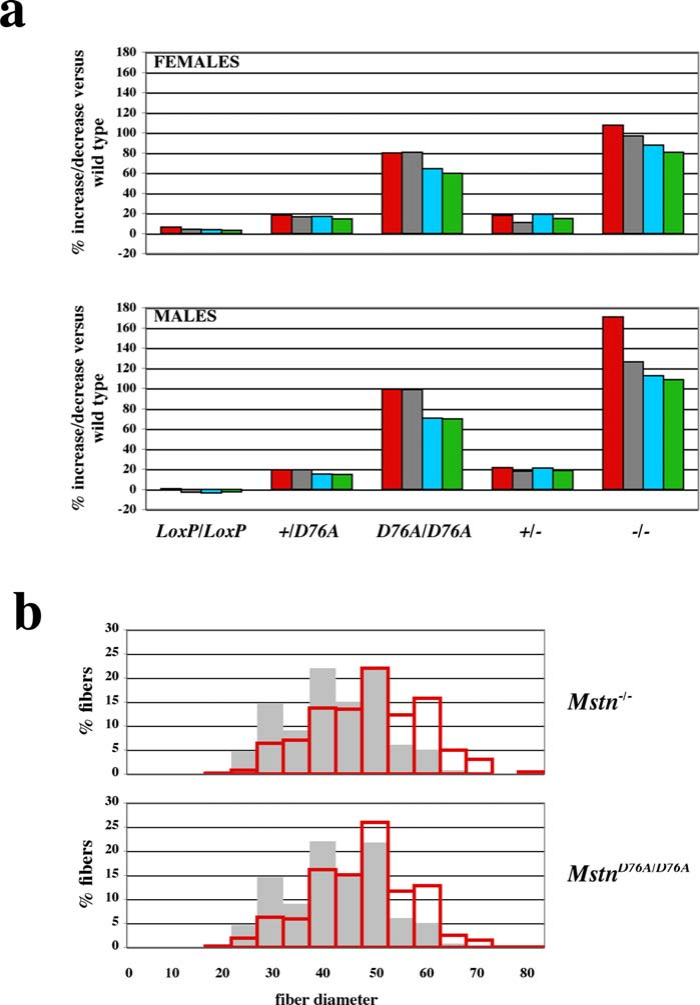
Analysis of muscles of mutant mice. (a) Muscle weight increases in *Mstn^D76A/D76A^* mice. Numbers represent percent increases relative to wild type mice and were calculated from the data shown in Table 1. Muscles analyzed were: pectoralis (red), triceps (gray), quadriceps (blue), and gastrocnemius (green). (b) Distribution of fiber diameters. Gray bars represent muscle fibers from wild type mice, and red bars represent muscle fibers from *Mstn^−/−^* and *Mstn^D76A/D76A^*.

As shown in Table 1 and Figure 2a, mice homozygous for the LoxP allele but wild type at aspartate 76 had muscle weights that were comparable to those of wild type mice. In contrast, mice carrying the D76A point mutation exhibited significant increases in muscle mass in all four skeletal muscles that were examined as well as in both males and females. Moreover, the effect of the point mutation was dose-dependent, with mice heterozygous for the mutation exhibiting increases of 14–19% and mice homozygous for the mutation exhibiting increases of 60–99% compared to wild type mice. This overall pattern was qualitatively similar to that observed in mice heterozygous or homozygous for a *Mstn* null allele; however, the effect of the point mutation was slightly reduced in magnitude compared to that of the null allele.

**Table 1:** Muscle weights (mg) of *Mstn* and *Tll2* mutant mice.

offspring	sex	n	pectoralis	triceps	quadriceps	gastrocnemius
*Mstn* ***^+/+^***	Female	11	51.5±1.6	74.5±1.2	144.8±3.7	101.6±2.3
*Mstn* ***^LoxP/LoxP^***	Female	10	54.7±1.9	77.5±1.4	150.1±3.9	104.6±2.6
*Mstn* ***^+/D76A^***	Female	12	61.0±1.1 a	86.6±2.0 a	169.3±3.3 a	116.2±1.8 a
*Mstn* ***^D76A/D76A^***	Female	10	92.7±4.3 a	134.4±4.5 a	238.1±9.3 a	162.3±5.7 a
*Mstn* ***^+/−^***	Female	12	60.8±1.3 b	82.5±1.8 b	172.1±4.0 a	116.5±3.2 b
*Mstn* ***^−/−^***	Female	10	106.9±4.0 a,c	146.6±4.7 a	271.9±9.6 a,c	183.4±4.7 a,d
*Tll2^−/−^*	Female	12	56.0±1.2 e	81.8±1.9 f	162.8±2.6 b	112.3±2.0 f
*Mstn* ***^+/+^***	Male	11	78.5±3.7	101.3±3.2	196.7±7.7	137.9±4.4
*Mstn* ***^LoxP/LoxP^***	Male	12	78.8±1.7	98.2±2.7	189.4±5.1	134.0±3.5
*Mstn* ***^+/D76A^***	Male	15	93.5±2.5 f	120.9±3.0 b	226.2±6.9 f	157.9±3.9 f
*Mstn* ***^D76A/D76A^***	Male	10	156.2±8.8 a	201.2±10.2 a	335.9±14.9 a	234.5±8.9 a
*Mstn* ***^+/−^***	Male	12	95.2±2.8 f	119.4±3.7 b	238.1±6.8 b	163.8±4.0 b
*Mstn* ***^−/−^***	Male	10	212.4±3.6 a,g	229.1±5.6 a,c	417.8±9.4 a,h	287.5±4.9 a,g
*Tll2^−/−^*	Male	15	83.1±1.1	106.1±1.6	213.1±2.9	149.1±1.1 e

a *p*<0.0001 vs. wild type;

b *p*<0.001 vs. wild type;

c *p*<0.05 vs. *Mstn* ***^D76A/D76A^***;

d *p*<0.01 vs. *Mstn* ***^D76A/D76A^***;

e *p*<0.05 vs. wild type;

f *p*<0.01 vs. wild type;

g *p*<0.0001 vs. *Mstn* ***^D76A/D76A^***;

h *p*<0.001 vs. *Mstn* ***^D76A/D76A^***

In previous studies, we showed that the increases in muscle mass seen in *Mstn* null mice result from a combination of increased fiber numbers and muscle fiber hypertrophy [2]. To determine whether the same is true for mice carrying the D76A point mutation, I carried out morphometric analysis of the gastrocnemius/plantaris muscles. As shown in Table 2 and Figure 2b, total fiber number was increased by 48% in *Mstn^−/−^* mice compared to wild type mice, and mean fiber diameter at the widest point of the muscles was increased by 16%, which would be predicted to result in an approximately 34% increase in cross-sectional area. Hence, consistent with what I reported previously [21], increases in both fiber number and fiber size appeared to contribute to the increase in mass seen in the gastrocnemius/plantaris muscles in *Mstn^−/−^* mice. Similarly, both muscle fiber number and mean fiber diameter were increased in the gastrocnemius/plantaris muscles of mice homozygous for the D76A point mutation, although the effects seen in *Mstn^D76A/D76A^* mice appeared to be slightly lower than those seen in *Mstn^−/−^* mice (Table 2 and Figure 2b).

**Table 2:** Morphometric analysis of gastrocnemius/plantaris muscles.

genotype	n	total fiber number	relative fiber number	mean fiber diameter (µm)	relative fiber diameter	relative cross-sectional area a
*Mstn* ***^+/+^***	5	8430±225	1.00	42.4±0.9	1.00	1.00
*Mstn* ***^D76A/D76A^***	5	12123±538 b	1.44	47.5±1.3 c	1.12	1.26
*Mstn* ***^−/−^***	3	12495±1138 d	1.48	49.0±0.8 b	1.16	1.34

a calculated as relative fiber diameter squared,

b *p*<0.001 vs. *Mstn* ***^+/+^***,

c *p*<0.01 vs. *Mstn* ***^+/+^***;

d *p*<0.05 vs. *Mstn* ***^+/+^***

Hence, the phenotype of *Mstn^D76A/D76A^* mice closely resembled the phenotype of *Mstn* null mice. Although these findings were consistent with the point mutation rendering the latent myostatin complex resistant to activation by BMP-1/TLD proteases, it was important to rule out the possibility that the point mutation caused increased muscling by decreasing myostatin expression levels. I first examined effects of the D76A point mutation on *Mstn* RNA levels by Northern analysis. As shown in Figure 1b, analysis of four different muscles showed that levels of *Mstn* mRNA were not decreased in muscles of *Mstn^D76A/D76A^* mice compared to wild type mice and, in fact, actually appeared to be even slightly increased. Next, I examined effects of the *D76A* mutation on circulating myostatin protein levels. Because I was unable to detect myostatin protein directly in serum samples by Western analysis using antibodies that we had prepared against recombinant myostatin protein, I partially purified the complex of myostatin propeptide and C-terminal dimer from serum by taking advantage of the ability of both the wild type and mutant complex to bind hydroxylapatite [12], [15]. Western analysis of the hydroxylapatite-bound material revealed that serum levels of both the propeptide and the C-terminal domain were dramatically increased in *Mstn^D76A/D76A^* mice compared to wild type mice (Figure 1c); in fact, in these experiments, the C-terminal domain was readily detectable in serum samples from *Mstn^D76A/D76A^* mice but only barely so in serum samples from wild type mice. Hence, even though the complex of mutant propeptide and C-terminal dimer appears to accumulate to high levels in *Mstn^D76A/D76A^* mice, the latent complex is unable to be activated, thereby resulting in increased muscle growth.

### Generation and characterization of *Tll2* mutant mice

The BMP-1/TLD family consists of four proteins, BMP-1, TLD, TLL-1, and TLL-2, encoded by three distinct genes (*Bmp1*, *Tll1*, and *Tll2*; for review, see ref. 22). All four of these proteases are capable of cleaving and activating the latent myostatin complex *in vitro* [12]. For two reasons, TLL-2 seemed to be an attractive candidate for the protease that might be important for regulating myostatin latency. First, unlike *Bmp1* and *Tll1*, *Tll2* is expressed during limb development specifically in skeletal muscle [23]. Second, although all four proteases are capable of cleaving the myostatin propeptide, the range of substrates on which TLL-2 is active appears to be much more narrow than those of other family members [22], raising the possibility that regulation of myostatin activity might be one of the primary functions of TLL-2 *in vivo*.

**Figure 3:**
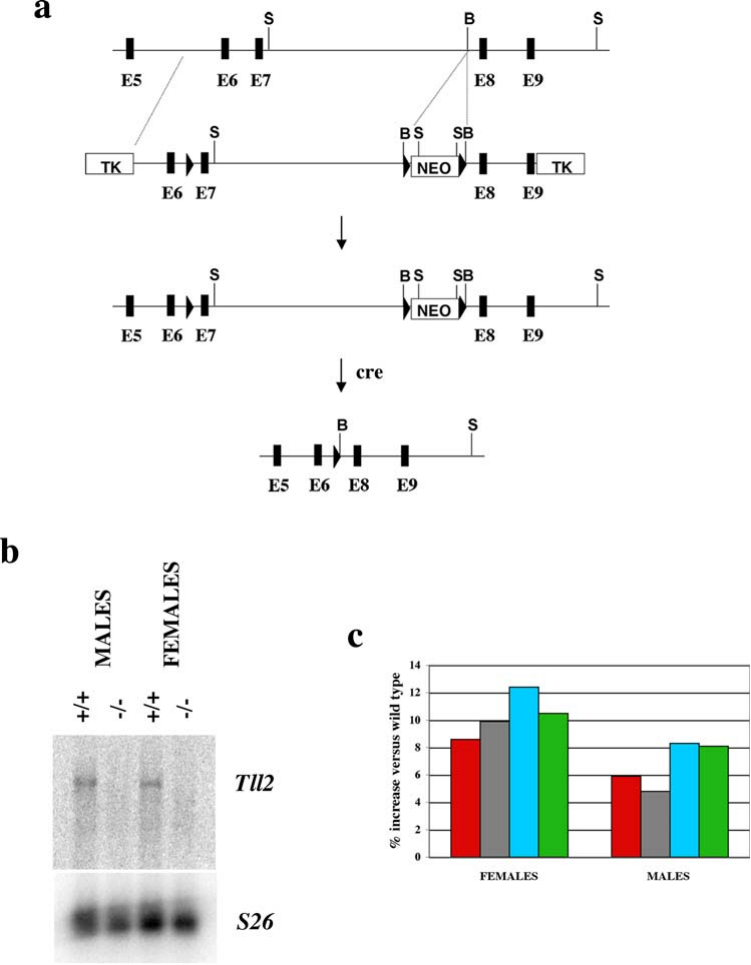
Generation and analysis of mice carrying a loss-of-function mutation in the *Tll2* gene. (a) Gene targeting strategy. Locations of exons 6–9 are shown as black boxes, and LoxP sites are denoted by triangles. (b) Northern analysis of *Tll2* expression levels. Twenty micrograms of poly A-selected brain RNA isolated from either wild type or *Tll2^−/−^* mice were electrophoresed, blotted, and hybridized with a *Tll2* probe corresponding to exons 1–3. The blot was re-hybridized with a probe for the S26 ribosomal protein to control for loading. (c) Muscle weight increases in *Tll2^−/−^* mice. Numbers represent percent increases relative to wild type mice and were calculated from the data shown in Table 1. Muscles analyzed were: pectoralis (red), triceps (gray), quadriceps (blue), and gastrocnemius (green).

To determine whether TLL-2 plays a role in regulating myostatin latency *in vivo*, I generated mice carrying a targeted deletion of the *Tll2* gene. The *Tll2* gene consists of 21 exons, with the protease domain being encoded in exons 4–8. As shown in Figure 3a, I generated a construct in which a LoxP site and a LoxP/neo cassette were introduced into introns 6 and 7, respectively. Following homologous recombination in embryonic stem cells and transfer of the targeted cells into blastocysts, several chimeric mice were obtained that transmitted the targeted allele through the germline. Offspring from the chimeric mice were then mated with EIIa-cre transgenic mice to generate mice in which exon 7 had been completely deleted. The deletion allele was then backcrossed six times onto a C57 BL/6 genetic background prior to analysis. Mice homozygous for the deletion were viable and fertile, and all analysis was carried out on 10-week old mice.

A deletion of exon 7 is predicted to remove approximately half of the protease domain of TLL-2 as well as cause a frameshift leading to a premature termination codon. Hence, this mutant allele would be expected to result in a severely truncated product lacking over 85% of the normal protein, including not only half of the protease domain but also all of the CUB and EGF domains. The presence of a premature termination codon near the 5′ end of the mRNA also raised the possibility that the mutant transcript might be subject to nonsense-mediated decay. To determine whether the mutant transcript was unstable, I compared RNA levels in brains of wild type and *Tll2^−/−^* mice. As shown in Figure 3b, the *Tll2* transcript was readily detected by Northern analysis of poly A-selected brain RNA prepared from wild type mice. In contrast, the deletion transcript was undetectable in brains of *Tll2^−/−^* mice, consistent with nonsense-mediated decay. Hence, the deletion of exon 7 appears to result not only in a severely truncated protein consisting of about half of the protease domain but also in greatly reduced mRNA levels and therefore likely results in a severe, if not complete, reduction in TLL-2 activity.

To determine whether TLL-2 may play a role in regulating myostatin activity, I measured effects of the *Tll2* mutation on muscle mass. As shown in Table 1 and Figure 3c, mice homozygous for the *Tll2* mutation exhibited slightly increased muscle weights compared to wild type mice. The increases in muscle weights in *Tll2^−/−^* mice were more pronounced in females, which exhibited statistically significant increases ranging from 9–12% depending on the specific muscle. Based on these results, TLL-2 appears to play at least some role in regulating muscle growth; however, the fact that the muscle mass increases seen in *Tll2^−/−^* mice were significantly lower than those seen in *Mstn^D76A/D76A^* mice implies that TLL-2 cannot be the sole protease involved in regulating myostatin latency *in vivo*.

## Discussion

Here, I have presented the results of genetic studies investigating the mechanisms by which myostatin activity is regulated *in vivo*. Previous studies have demonstrated that following processing of the myostatin precursor protein by furin proteases, the propeptide remains non-covalently bound to the mature C-terminal dimer and maintains it in a latent, inactive state [12], [15], [16]. This latent complex of the propeptide and C-terminal dimer also appears to represent the predominant form of myostatin that circulates in the blood [17], [18]. A major question regarding the mechanisms by which myostatin regulates muscle growth *in vivo* is how myostatin is activated from this latent state. In an earlier study, we demonstrated that members of the BMP-1/TLD family of metalloproteases are capable of cleaving the myostatin propeptide and thereby activating the latent myostatin complex *in vitro* [12]. In order to determine whether this activation mechanism operates *in vivo*, I analyzed the effect of blocking this pathway in mice.

We showed previously that members of the BMP-1/TLD family can cleave the myostatin propeptide immediately N-terminal to aspartate residue 76. Furthermore, we showed that changing this aspartate to alanine (D76A) had no effect on the ability of the propeptide to form a latent complex with the mature myostatin C-terminal dimer but rendered the propeptide completely resistant to proteolysis by BMP-1/TLD proteases *in vitro* [12]. Here, I have analyzed the effect of introducing this D76A point mutation into the endogenous *Mstn* gene by homologous targeting in mice. I have presented data showing that mice homozygous for this *D76A* mutation exhibit significant increases in muscle mass and, as in the case of mice completely lacking myostatin, that the increases in *Mstn^D76A/D76A^* mice result from a combination of increased fiber numbers and increased fiber sizes. Remarkably, these mice exhibit significant increases in muscle mass despite the fact that the circulating levels of myostatin protein in these mice are dramatically increased compared to wild type mice. Hence, in *Mstn^D76A/D76A^* mice, myostatin appears to exist in a latent state that, for the most part, cannot be activated, and as a result, this latent complex accumulates to high levels. Based on the enhanced muscling seen in these mutant mice, it seems clear that proteolysis of the propeptide is the major mechanism by which latent myostatin is activated *in vivo*; however, the fact that the increases in muscle mass seen in these mice are slightly lower than those seen in mice completely lacking myostatin implies that this cannot be the sole mechanism for activating the latent complex.

The BMP-1/TLD family consists of four proteins, each of which is capable of cleaving and activating the latent myostatin complex *in vitro* [12]. Based on its expression pattern and substrate specificity, one of these family members, TLL-2, appeared to be an attractive candidate for the protease that might be responsible for activating latent myostatin *in vivo*. To investigate this possibility, I generated and analyzed mice carrying a targeted mutation in the *Tll2* gene. Mice homozygous for the *Tll2* mutation exhibit increases in muscle mass, but these increases are relatively small compared to those seen in either *Mstn^–/–^* or *Mstn^D76A/D76A^* mice. These results suggest either that TLL-2 plays a relatively minor role in activating latent myostatin *in vivo* or that its function is redundant with those of other members of this protease family.

Clearly, the next step will be to examine the potential roles of the other members of the BMP-1/TLD family in regulating myostatin latency. In this regard, it will be important to investigate the roles of both BMP-1/TLD and TLL-1, as *Bmp1* has been shown to be expressed in skeletal muscle during development in an overlapping pattern with *Tll2*, and both *Bmp1* and *Tll1* have been shown to be expressed in adult skeletal muscle [23]–[25]. Given that genetic studies have shown that complete loss of either BMP-1/TLD or TLL-1 causes perinatal or embryonic lethality in mice [26], [27], analysis of the roles of these proteases in regulating muscle mass will require the generation of mice in which each of these proteases can be eliminated in a tissue-specific manner.

The elucidation of the precise roles played by each of these proteases will be essential for targeting this regulatory mechanism for the development of drugs capable of blocking myostatin activity. These studies are particularly important in that the relevant protease or proteases would represent an attractive target for small molecule drug screening. Although several biologics have been identified that can target myostatin activity and promote muscle growth, the development of small molecule inhibitors capable of mimicking these effects has been hampered by the general paucity of suitable targets for drug screening. The studies presented here should provide a strong impetus for further pursuing these proteases for the development of agents capable of promoting muscle growth in clinical settings where increasing muscle strength may be beneficial.

## Methods

Targeting constructs were generated from phage clones isolated from a 129 SvJ genomic library [2]. R1 embryonic stem cells were kindly provided by A. Nagy, R. Nagy, and W. Abramow-Newerly. Blastocyst injections of targeted clones were carried out by the Johns Hopkins Transgenic Core Facility. All mice, including *Mstn^−/−^* mice [2], were backcrossed at least 6 times onto a C57 BL/6 background prior to analysis. All analysis was carried out on 10-week old mice. All animal experiments were carried out in accordance with protocols that were approved by the Institutional Animal Care and Use Committee at Johns Hopkins University School of Medicine.

For measurement of muscle weights, individual muscles were dissected from both sides of the animal, and the average weight was calculated. For morphometric analysis, the gastrocnemius and plantaris muscles were sectioned to their widest point using a cryostat, and fiber diameters were measured from hematoxylin and eosin stained sections. Fiber measurements were carried out on 250 fibers per animal, and mean fiber diameters were calculated for each animal. For plotting the distribution of fiber sizes, all data for a given genotype were pooled.

For analysis of myostatin protein, the myostatin latent complex was partially purified based on its ability to bind hydroxylapatite [15]. Mouse serum was diluted five-fold with 50 mM Tris pH 7.4 and bound to hydroxylapatite. After washing the column with 50 mM sodium phosphate pH 7.2, the myostatin latent complex was eluted with 200 mM sodium phosphate pH 7.2. The eluate was dialyzed, lyophilized, and subjected to Western analysis.
